# Supplementary figures and images for: Salivary proteomics in monitoring the therapeutic response of canine oral melanoma
Source: PLoS One. 2021 Aug 19;16(8):e0256167. doi: 10.1371/journal.pone.0256167 (PMC8376060; doi:10.1371/journal.pone.0256167)

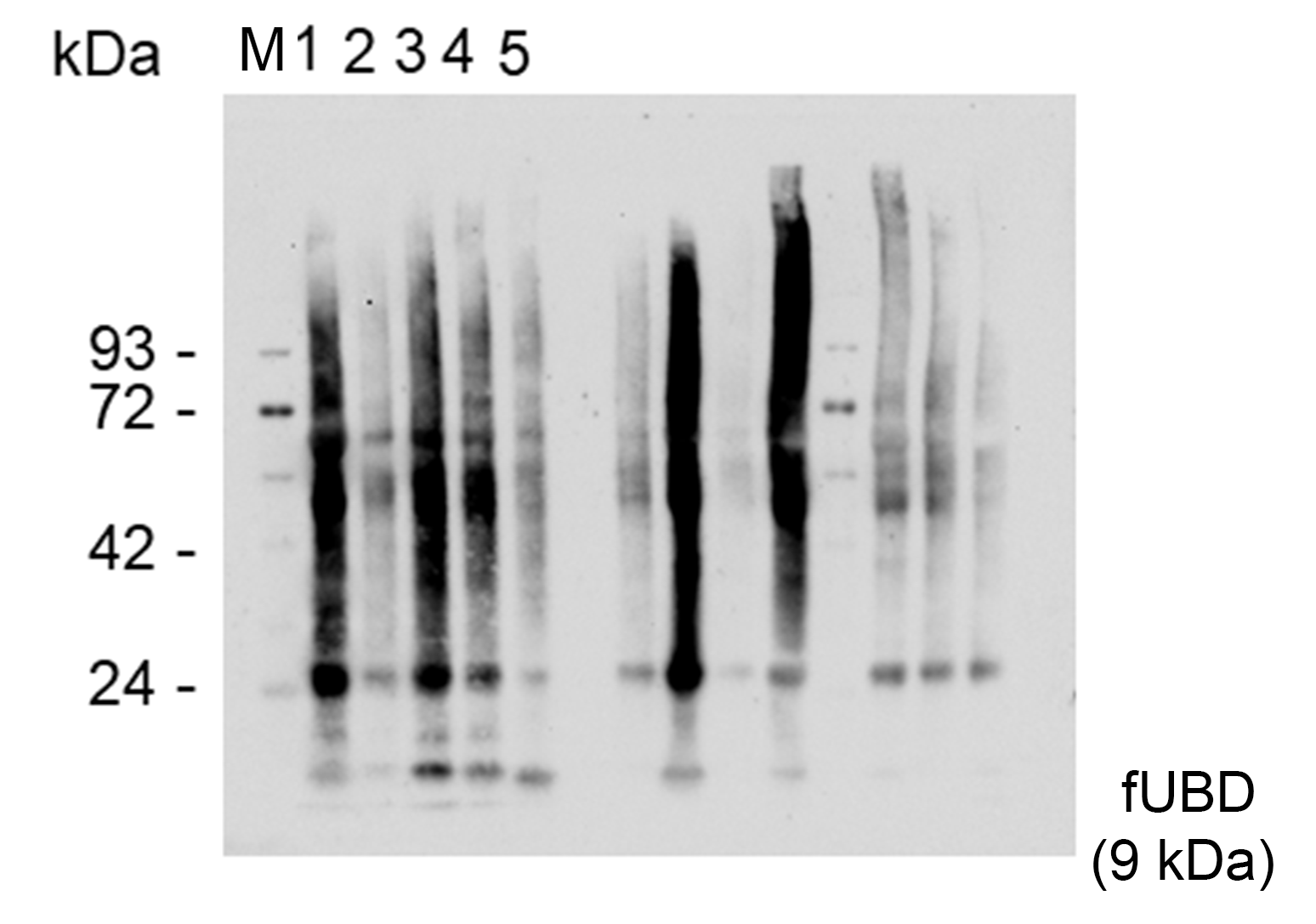

Supplement: S1 Fig — Lane M, prestained protein ladder marker; lane 1, pre-surgery (PreS); lane 2, post-surgery (PostS); lane 3, after treating with chemotherapy drug once; lane 4, after treating with chemotherapy drug twice; lane 5, after treating with chemotherapy drug 3 times. (TIF) [file pone.0256167.s001.tif]

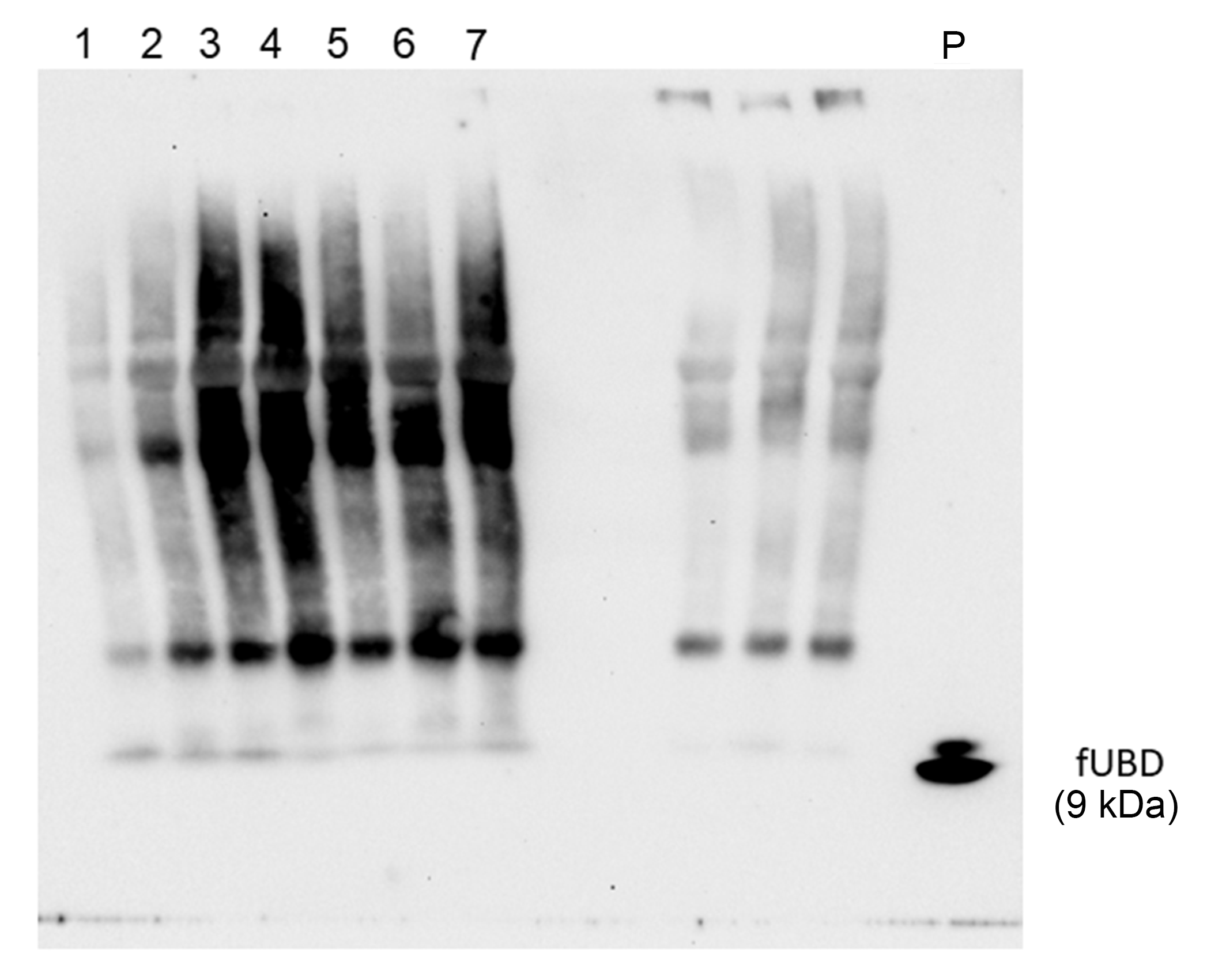

Supplement: S2 Fig — Lane 1, pre-surgery (PreS) of patient No. ….; lane 2, post-surgery (PostS); lane 3, after treating with chemotherapy drug once; lane 4, after treating with chemotherapy drug twice; lane 5, after treating with chemotherapy drug 3 times; lane 6, after treating with chemotherapy drug 4 times; lane 7, after treating with chemotherapy drug 5 times; lane P, positive control. (TIF) [file pone.0256167.s002.tif]

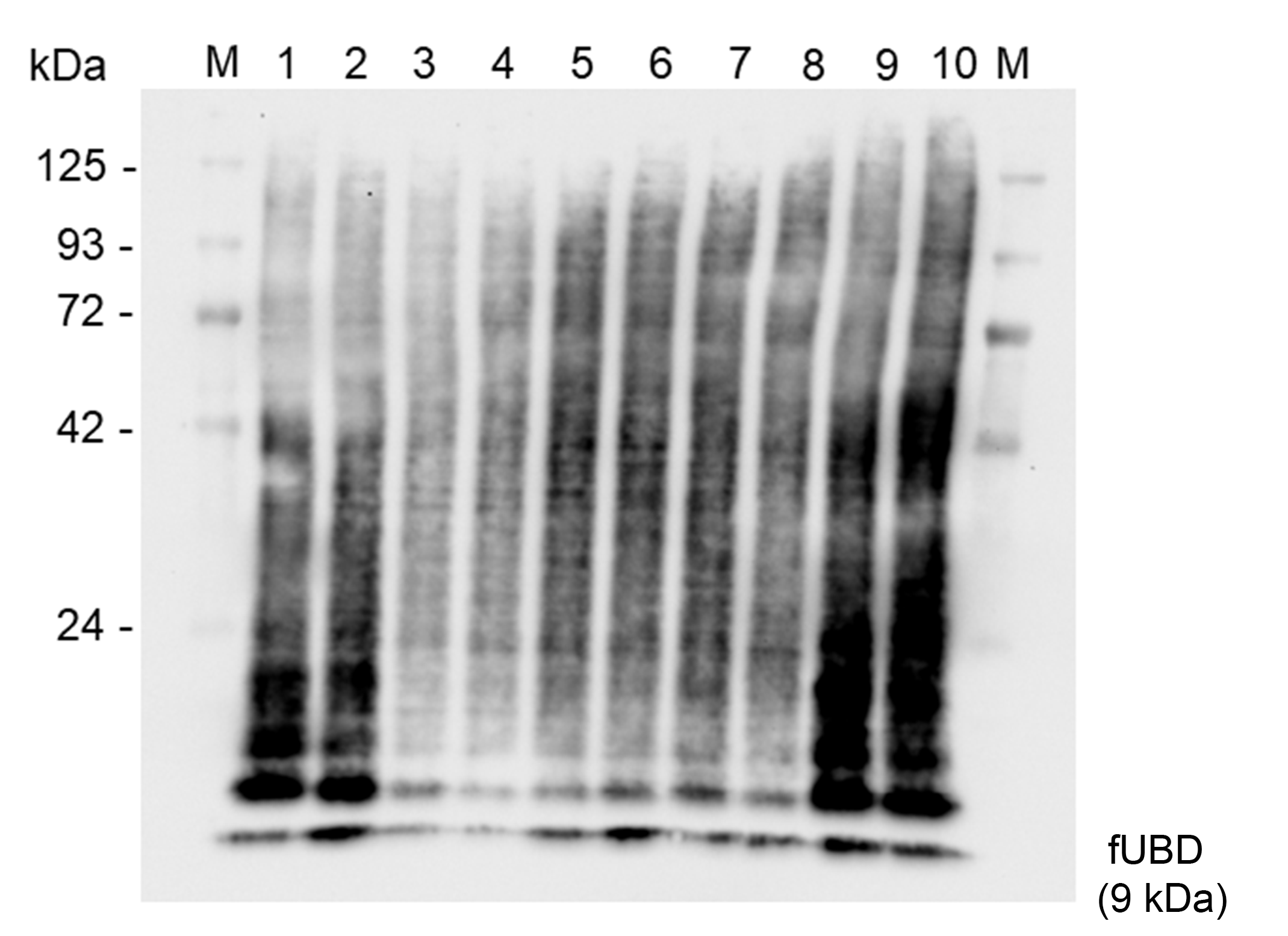

Supplement: S3 Fig — Lane M, prestained protein ladder marker; lane 1, pre-surgery (PreS); lane 2, post-surgery (PostS); lane 3, after treating with chemotherapy drug twice; lane 4, after treating with chemotherapy drug 3 times; lane 5, after treating with chemotherapy drug 4 times; lane 6, after treating with chemotherapy drug 6 times; lane 7, check-up after treating with chemotherapy drug 2 months; lane 8, check-up after treating with chemotherapy drug 4 months; lane 9, check-up after treating with chemotherapy drug 6 months; lane 10, check-up after treating with chemotherapy drug 8 months. (TIF) [file pone.0256167.s003.tif]

kDa M 1 2 3 4 5

93 -  
72 -  
42 -  
24 -

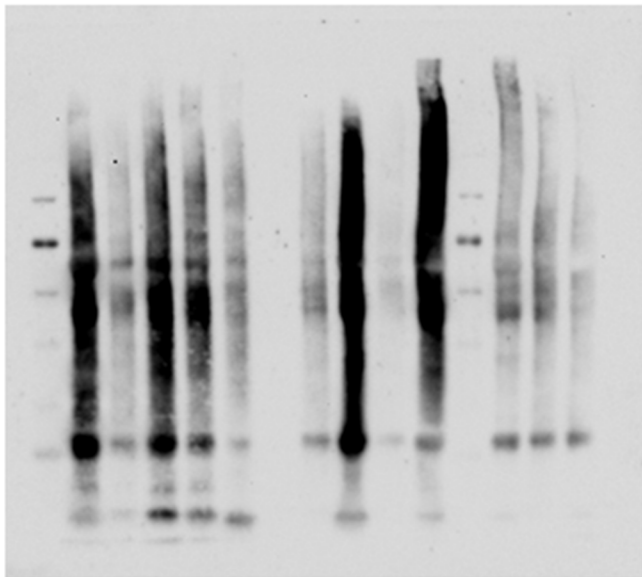

fUBD  
(9 kDa)

1 2 3 4 5 6 7

P

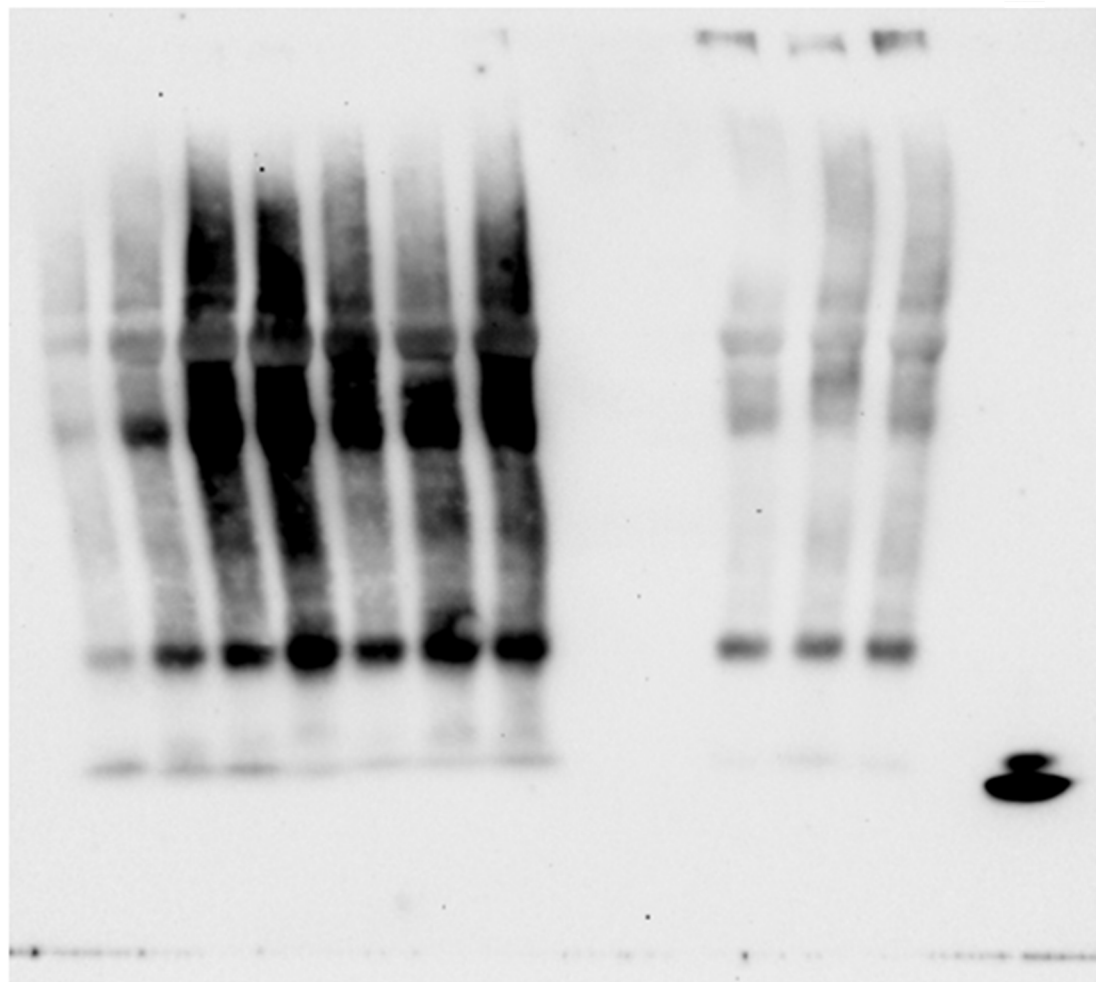

fUBD  
(9 kDa)

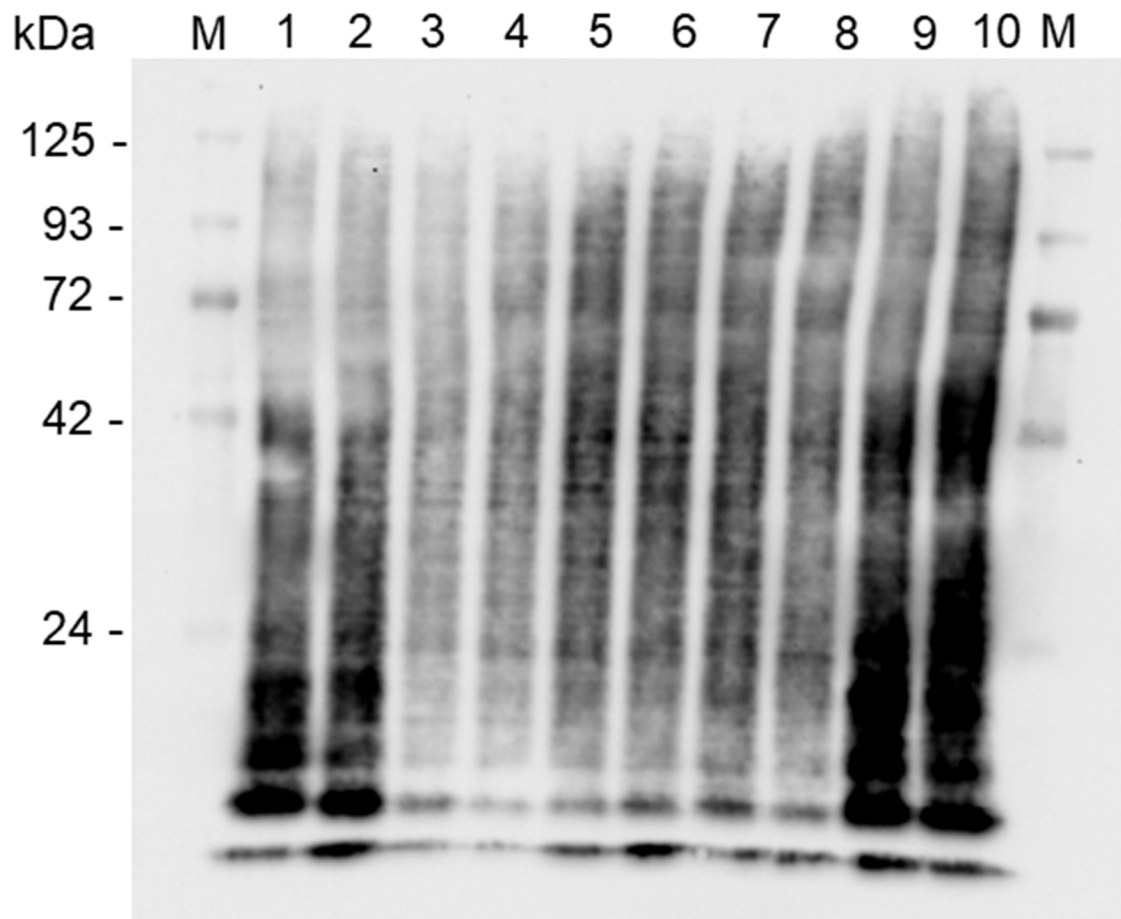

fUBD  
(9 kDa)

Supplement: S1 Raw images — (PDF) [file pone.0256167.s007.pdf]
